# Genome Analysis of *Pseudomonas viciae* G166 Conferring Antifungal Activity in Grapevine

**DOI:** 10.3390/jof10060398

**Published:** 2024-05-31

**Authors:** Xiaoshu Jing, Ling Su, Xiangtian Yin, Yingchun Chen, Xueqiang Guan, Dongyue Yang, Yuxia Sun

**Affiliations:** Shandong Academy of Grape, Shandong Academy of Agricultural Sciences, Jinan 250100, China; johncy@126.com (X.J.); susulingzi@126.com (L.S.); yinxiangtian@shandong.cn (X.Y.); spring024@126.com (Y.C.); guanxq90@126.com (X.G.)

**Keywords:** grape white rot disease, comparative genome analysis, non-ribosomal lipopeptides, biocontrol agents (BCAs)

## Abstract

Grapevine (*Vitis vinifera*) is one of the major economic fruit crops but suffers many diseases, causing damage to the quality of grapes. Strain G166 was isolated from the rhizosphere of grapevine and was found to exhibited broad-spectrum antagonistic activities against fungal pathogens on grapes in vitro, such as *Coniella diplodiella*, *Botrytis cinerea,* and *Colletotrichum gloeosporioides*. Whole-genome sequencing revealed that G166 contained a 6,613,582 bp circular chromosome with 5749 predicted coding DNA sequences and an average GC content of 60.57%. TYGS analysis revealed that G166 belongs to *Pseudomonas viciae*. Phenotype analysis indicated that *P. viciae* G166 remarkably reduced the severity of grape white rot disease in the grapevine. After inoculation with *C. diplodiella*, more H_2_O_2_ and MDA accumulated in the leaves and resulted in decreases in the Pn and chlorophyll content. Conversely, G166-treated grapevine displayed less oxidative damage with lower H_2_O_2_ levels and MDA contents under the pathogen treatments. Subsequently, G166-treated grapevine could sustain a normal Pn and chlorophyll content. Moreover, the application of *P. viciae* G166 inhibited the growth of mycelia on detached leaves and berries, while more disease symptoms occurred in non-bacterized leaves and berries. Therefore, *P. viciae* G166 served as a powerful bioagent against grape white rot disease. Using antiSMASH prediction and genome comparisons, a relationship between non-ribosomal peptide synthase clusters and antifungal activity was found in the genome of *P. viciae* G166. Taken together, *P. viciae* G166 shows promising antifungal potential to improve fruit quality and yield in ecological agriculture.

## 1. Introduction

Grapevine (*Vitis vinifera*) is one of the major broadly cultivated economic fruit crops worldwide because of its fleshy berries and the wine produced as its end-product. However, grapevines are exposed to many diseases caused by fungi, bacteria, and viruses [1], which can reduce their fruit productivity and affect the quality of both grapes and wine by altering the biochemistry and composition of the grapes, causing significant economic damage to vineyards and wineries. Synthetic microbicides are used in the suppression of phytopathogens in agriculture but have led to harmful consequences for the environment and human health. Ecofriendly sustainable biological agents are promising in solving the ecological issues associated with the overuse of agrochemicals in agriculture. Plant-growth-promoting rhizobacteria (PGPRs) are a better alternative, as they reinforce crop resistance against pathogens through direct and indirect mechanisms [2,3,4]. Many beneficial microorganisms have been discovered among bacteria of several genera, e.g., *Bacillus*, *Burkholderia*, *Agrobacterium*, and *Pseudomonas* [5,6,7,8]. *Pseudomonas* is a universal genus in different ecological niches and exhibits a versatile metabolic capacity [9]. A number of *pseudomonas* strains function as PGPRs to protect plants from various pathogens and/or stimulate plant growth [10,11,12,13,14,15]. For example, *P*. *chlororaphis* [11] and *P. protegens* [10,12] are non-pathogenic biocontrol agents, while several strains of *P. stutzeri* [14,15] show strong plant-growth-promoting activities. Bioactive secondary metabolites are the major defense mechanism against pathogenic microorganisms. *Pseudomonas* strains produce different secondary compounds with antifungal activity, such as 2,4-diacetylphloroglucinol (2,4-DAPG) [12,16], pyrrolnitrin (PRN) [17], pyoluteorin (PLT) [18], and non-ribosomal peptides (NRPs) [19,20,21,22,23,24,25,26,27,28]. Non-ribosomal lipopeptides (NRLPs) are oligopeptides that are N-terminally acylated with a fatty acid and are subdivided into linear lipopeptides (LLPs) and cyclic lipopeptides (CLPs) [9]. Based on the four secondary metabolites—DAPG, PLT, PRN, and CLPs—the *pseudomonas* genus is divided into three groups [19]: Group I, *Pseudomonas* spp., which produces all four types of antimicrobial compounds. Group II only produces DAPG but not PLT, PRN, or CLPs. Group III produces CLPs but not DAPG, PLT, or PRN. Lipopeptides of many isolated *Pseudomonas* spp. have shown a considerable structural diversity and play an important role in biocontrol abilities, such as nunamycin and nunapeptin in *P. fluorescens* In5 [20,21], brasmycin and braspeptin in *Pseudomonas* sp. 11K1 [19], thanamycin and thanapeptin in *Pseudomonas* sp. SH-C52 [22,23], cormycin and corpeptin in *P. corrugata* CFBP5454 [24,25], and orfamide in *P. protegens* [26,27,28]. Driven largely by the vast amounts of genomic information, comparative genomics is used to further understand the genome evolution and gene function and even to screen new antimicrobial compounds with the conserved clusters.

Strain G166 was isolated from the rhizosphere of grape. Based on the Type (Strain) Genome Server (TYGS) for whole-genome-based taxonomic analysis, G166 was identified in *Pseudomonas viciae*. Its in vivo potential to control white rot disease in grapevine was evaluated, and a comparative genome analysis of all publicly available sequenced *P. viciae* strains was performed. Overall, this study is the first to exploit a potential *pseudomonas viciae* biocontrol agent for grape production. In silico genome mining revealed that *P. viciae* G166 could be a good candidate for discovering new NRLPs, and it might increase the possibility of discovering new bioactive products. Furthermore, the *P. viciae* G166 strain originally isolated from the rhizosphere of grapevine may have greater potential to be sustained within the host.

## 2. Materials and Methods

### 2.1. Strains, Plant Material, and Growth Conditions

*P. viciae* G166 was isolated from grape rhizosphere soil in Jinan city, Shandong Province, China, in March 2021. Ten grams of soil was suspended in 90 mL of sterilized saline and shaken at 200 rpm for 10 min. Then, 1.0 mL of this suspension was serially diluted up to 10^−8^, and the dilutions were spread on King’s B medium (KB) and incubated at 28 °C overnight. Isolated colonies were further screened for their ability to suppress *C. diplodiella*. *P. viciae* G166 was found to exhibit an antagonistic effect against *C. diplodiella*. A PCR amplification of 16S rRNA of the bacteria was performed with the primers 27F (5′-AGAGTTTGATCMTGGCTCAG-3′) and 1492R (5′-GGTTACCTTGTTA CGACTT-3′) [29]. The sequenced 16S rRNA gene fragment was compared with the NCBI nucleotide database using Blastn (https://blast.ncbi.nlm.nih.gov/Blast.cgi) to determine the closest taxonomic relatives.

*P. viciae* G166 was grown in KB medium at 28 °C overnight with shaking at 200 rpm. The fungi (*C. diplodiella*, *B. cinerea,* and *C. gloeosporioides*) were grown on PDA plates at 28 °C.

Seedlings of the Muscat grape ‘Shine Muscat’ *(Vitis labrusca × V. vinifera)* were planted in 4.5 L pots and fertilized with half-strength Hoagland’s nutrient solution every 2 weeks at 20–25 °C. The plants were cultured 30–40 cm high for 2 months before the experiments. Six plantlets were used for each treatment. The leaves were used for biocontrol activity analysis and measurements of the chlorophyll contents, net photosynthetic rate (Pn), MDA contents, and H_2_O_2_. Berries from five-year-old ‘Shine Muscat’ grapes were picked for biocontrol activity analysis in the maturity stage. Ten leaves/berries were used for each treatment, and the experiments were independently repeated three times.

### 2.2. In Vitro Antifungal Activity for Inhibition of Mycelium Growth

The fungal growth inhibition by *P. viciae* G166 was demonstrated using the dual culture method on PDA plates [12]. The fungal test strains were *C. diplodiella*, *B. cinerea*, and *C. gloeosporioides.* A 1 cm agar plug was placed in the middle of the PDA plates, and 10 μL of *P. viciae* G166 that was cultured overnight was inoculated on the PDA plates in triplicate. Plates inoculated with the plug were used as the control. The plates were incubated for 5 days at 28 °C in the dark. The results were expressed by measuring the diameter of the mycelium growth inhibition zone compared to the control plates. The percentage of growth inhibition was calculated using the following equation: *n* = [(diameter_control_ − diameter_inhibition_)/diameter_control_] × 100, where the diameter of inhibition is the colony diameter of the treated fungi between the two bacteria. The values were recorded as the means of four replicates, and each experiment was repeated three times.

### 2.3. Genome Sequencing, Assembly, and Putative Secondary Metabolite Clusters Analysis

Genomic DNA was extracted from cells cultured overnight using a QIAamp^®^ DNA Mini Kit (Qiagen, Valencia, CA, USA) according to the manufacturer’s instructions. The whole genome was sequenced using the PacBio Sequel platform (Pacific Biosciences Inc., Menlo Park, CA, USA) and Illumina NovaSeq PE150 (Illumina, San Diego, CA, USA), and the filtered subreads were assembled with SMRT Link v5.0.1 [30]. The assembly results were integrated with the CISA software, and the gapclose software was used to fill the gap in the preliminary assembly results. The final assembly result was obtained by removing identical lane pollutions by filtering the reads with low sequencing depths (less than 0.35 in average depth). The functions of genes were predicted by GO (Gene Ontology), KEGG (Kyoto Encyclopedia of Genes and Genomes), COG (Clusters of Orthologous Groups), NR (Non-Redundant Protein Database), TCDB (Transporter Classification Database), and Swiss-Prot. Transfer RNA (tRNA) genes were predicted using tRNAscan-SE. Ribosome RNA (rRNA) genes were analyzed using rRNAmmer. Small nuclear RNAs (snRNAs) were predicted using the Rfam database (http://Rfam.sanger.ac.uk/). CRISPR Finder was used for CRISPR identification. A genome overview was created by Circos to show the annotation information [31]. The secondary metabolism gene clusters were analyzed using the antiSMASH bacteria online version (https://antismash.secondarymetabolites.org/#!/start).

### 2.4. Phylogenetic Tree Construction

The evolutionary position of *P. viciae* G166 was determined by 16S rDNA gene sequence analysis and whole-genome-based taxonomic analysis. A phylogenetic tree was constructed based on the 16S rDNA gene sequences of *P. viciae* G166 and the other *Pseudomonas* type strains from NCBI. A phylogenetic tree of the *Pseudomonas* strains was constructed using MEGA 7 through the neighbor-joining method with 1000 bootstrap replicates [32].

Whole-genome-based taxonomic analysis was conducted using the Genome BLAST Distance Phylogeny approach (GBDP) by uploading genome sequence data to the Type (Strain) Genome Server (TYGS), a free bioinformatics platform accessible at https://tygs.dsmz.de [33]. The completed sequences of the type strains were taken from NCBI. The GenBank accession numbers of the type strains, submitted to the TYGS, were as follows: *P. aeruginosa* DSM 50071 (CP012001.1), *P. alvandae* SWRI17 (CP077080.1), *P. bijieensi*s L22-9 (CP048810.1), *P. brassicacearum* subsp. *brassicacearum* NFM421 (CP002585.1), *P. chlororaphis* ATCC 9446 (CP118151.1), *P. protegens* CHA0 (CP003190.1), *P. putida* ATCC 12633 (CP101910.1), *P. rhizophila* S211 (CP024081.1), *P. syringae* DC3000 (AE016853.1), *P. viciae* 11K1 (CP035088.1), and *P. zarinae* SWRI108 (CP077086.1).

### 2.5. Comparative Genomics Analysis

For the comparative genomics analysis, the genome sequences of *P. viciae* G166 were compared to those of *P. viciae* 11K1, *P. viciae* B21-062, *P. viciae* YsS1, *P. brassicacearum* subsp. *brassicacearum* NFM421, *P. bijieensis* L22-9, and *P. zarinae* SWRI108 using the MAUVE comparison software [34]. Complete genome sequences of all seven *Pseudomonas* strains were aligned and visualized in the progressive mode using MAUVE.

Additionally, a circular chromosomal map of all the genomes was generated using BLAST Ring Image Generator (BRIG) version 0.95 [35] to compare the genomes of the six closely related *Pseudomonas* species, with that of *P. viciae* G166 used as a reference.

### 2.6. Phenotype Analysis of the Biocontrol Activity of P. viciae G166 against C. diplodiella

The pathogenic strain *C. diplodiella* was grown on PDA plates at 28 °C before being used as an inoculation on leaves (PDA plugs with fungi from 7-day-old culture) and on berries (spore suspension from 4-week-old culture) [36]. The detached leaves were subjected to surface sterilization, and the berries were washed in 10% sodium hypochlorite for 1 min and then 75% ethanol for 1 min, followed by washing with distilled water three times. The detached leaves and berries were pre-incubated with the bacteria at a concentration of OD600 = 0.3 (~10^8−9^ CFU/mL).

After inoculation with the bacteria for 24 h, the detached leaves and berries were inoculated with the pathogen as follows. For inoculation of the detached leaves, the upper epidermal layer of the leaf was injured with a sterile needle for wound inoculation. Then, PDA plugs (1 cm diameter) with fungal mycelia were inoculated on the wounded sites of the leaves. Sterile agar plugs were used as the negative control. Wetted cotton swabs were placed in Petri dishes to maintain humidity. For inoculation on the detached berries, 10 µL of spore suspension (10^6^ spores/mL) was microinjected on the berries. Sterile water was used as the negative control. The leaves and the berries were incubated at 28 °C with 80% RH. Ten biological replicates were performed for each treatment, and the experiments were independently repeated three times.

For the bacteria-treated plants, each leaf of one-year-rooted plants was incubated with a *C. diplodiella*-germinated spore suspension (10^5^ conidia/mL) after 7 days of incubation with the bacteria at a concentration of OD600 = 0.3 (~10^8−9^ CFU/mL). For the non-bacterized treatment, each leaf of one-year-rooted plants was incubated with a *C. diplodiella*-germinated spore suspension (10^5^ conidia/mL). Additionally, the seedlings treated with sterile water or *P. viciae* G166 were used as negative controls. After 14 days, the 3rd–5th mature leaves were used for measurements of the chlorophyll contents, Pn, MDA contents, and H_2_O_2_. The chlorophyll contents were analyzed as described [37]. Pn was measured using a CIRAS-3 Portable Photosynthesis System (Hansatech Instruments Ltd., Norfolk, UK). The 3rd and 4th leaves were harvested, quickly frozen in liquid nitrogen, and used for measurements of the MDA contents and H_2_O_2_ using a SpectraMax^®^ Mini Multi-Mode Microplate Reader (Molecular Devices GmbH, Munich, Germany). The MDA contents were determined using an MDA Assay Kit (Solarbio, Beijing, China). The H_2_O_2_ levels were determined using a Micro H_2_O_2_ Assay Kit (Solarbio, Beijing, China). The means ± SD consisted of a pool of from 5 to 6 plantlets.

### 2.7. Statistical Analysis

Statistical analyses were performed using SPSS Version 21.0 (IBM Corp., Armonk, NY, USA) and GraphPad prism 8.0 (GraphPad Software, Inc., La Jolla, CA, USA). A *p*-value of <0.05 and a 95% confidence interval (CI) were considered statistically significant.

## 3. Results

### 3.1. Antagonistic Activity of P. viciae G166

**Figure 1:**
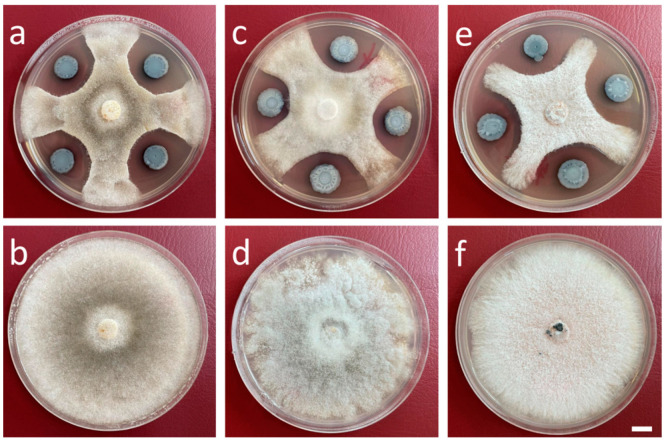
The antifungal activity of *P. viciae* G166. The antifungal acvitity of *P. viciae* G166 on *Coniella diplodiella* (**a**), *Botrytis cinerea* (**c**), and *Colletotrichum gloeosporioides* (**e**). (**b**,**d**,**f**) show pathogen cultures of *C. diplodiella* (**b**), *B. cinerea* (**d**), and *C. gloeosporioides* (**f**). Scale bar: 1 cm.

In comparison to the control, the *P. viciae* G166 strain was able to inhibit the mycelium growth of *C. diplodiella*, *B. cinerea,* and *C. gloeosporioides* (Figure 1). Strong inhibition zones appeared from the edge of the bacterial colony to the mycelium of *C. diplodiella*, *B. cinerea,* and *C. gloeosporioides,* with sizes of 6.65, 5.36, and 7.48 mm. Compared to the control plates, the diameters of the mycelium growth of *C. diplodiella*, *B. cinerea,* and *C. gloeosporioides* were inhibited by 54.98%, 53.45%, and 55.69%, respectively.

The results showed that *P. viciae* G166 exhibited antifungal activity against *C. diplodiella*, *B. cinerea,* and *C. gloeosporioides*.

### 3.2. Identification and Genomic Features of P. viciae G166

**Figure 2:**
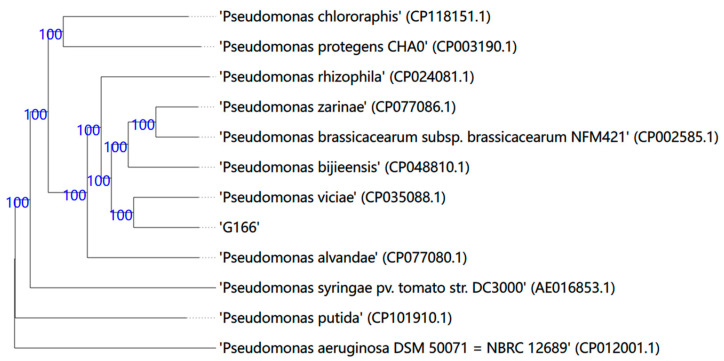
Phylogenetic tree generated by TYGS based on Genome BLAST Distance Phylogeny distances calculated from genome sequences. *P. aeruginosa* DSM50071 was used as the outgroup organism. Branch support was inferred from 100 pseudo-bootstrap replicates each. The trees were rooted at the midpoint.

Strain G166 is a Gram-negative bacterium of the order Pseudomonadales and class Gammaproteobacteria. A phylogenetic tree for the type strains based on 16S rDNA sequences analysis indicated that G166 was identified as a genus in the *Pseudomonas* group (Supplementary Figure S1). Among type strains, G166 shares a more recent common ancestor with *P. viciae* strains. The phylogenomic tree constructed based on TYGS for the whole-genome-based taxonomic analysis revealed the relationship between G166 and the closely related type strains (Figure 2). The G166 strain and type train *P. viciae* 11K1 formed a monophyletic group, indicating that G166 was identified as a *Pseudomonas viciae*.

**Figure 3:**
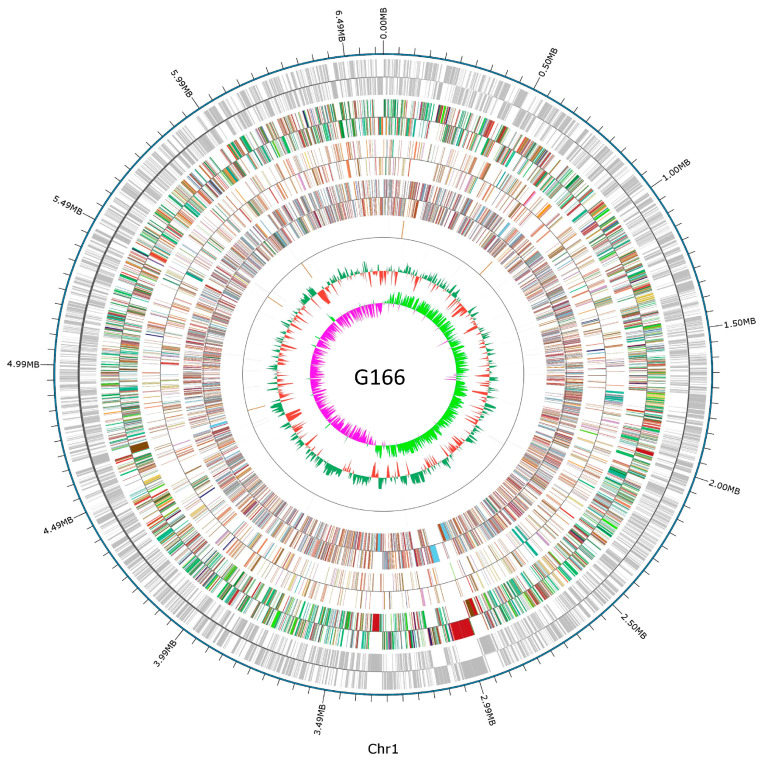
Graphical map of the chromosome. The outer scale is genomic position in kb. Circles range from 1 (outer circle) to 7 (inner circle): Circle 1, predicted protein-coding sequences (CDSs); Circle 2–4, distributions of function genes by COG (KOG), KEGG, and GO, respectively; Circle 5, distributions of ncRNA genes; Circle 6, GC content showing deviations from the average; Circle 7, GC skew (G − C/G + C).

The genome sequence of *P. viciae* G166 consisted of 6,613,582 bp with a G + C content of 60.57% (Figure 3, Table 1). In Figure 3, the circles range from 1 (outer circle) to 7 (inner circle): Circle 1 indicates predicted protein-coding sequences (CDSs); Circles 2–4 indicate distributions of function genes by COG (KOG), KEGG, and GO, respectively; Circle 5 indicates the distributions of ncRNA genes; Circle 6 indicates the GC content, showing deviations from the average; and Circle 7 indicates the GC skew (G − C/G + C). In total, 5880 genes were predicted, of which 5749 were function-annotated (Table 1). There were 67 predicted tRNAs, 16 predicted rRNAs, 17 predicted sRNAs, and 5 predicted CRISPs in G166 (Table 1).

### 3.3. Genome Comparisons among Pseudomonas spp.

**Figure 4:**
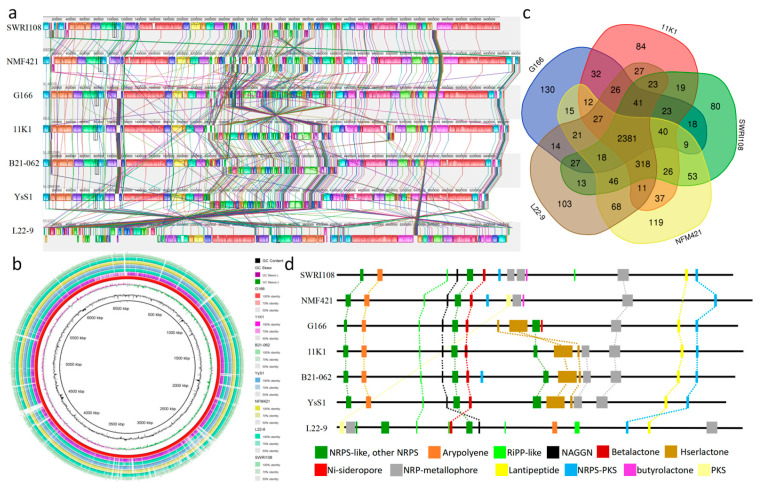
Comparison of *P. viciae* G166 genome sequences against other *Pseudomonas* genome sequences. (**a**) Synteny analysis of MAUVE progressive alignment using MAUVE. Boxes with the same color indicate syntenic regions. Boxes below the horizontal strain line indicate inverted regions. Rearrangements are shown by colored lines. Scale is in nucleotides. (**b**) Circular diagram illustrating the nucleotide similarity between the *Pseudomonas* genome sequences with BRIG 0.95. (**c**) Venn diagram of *P. viciae* G166, *P. viciae* 11K1, *P. viciae* B21-062, *P. brassicacearum* subsp. *brassicacearum* NFM421, *P. bijieensis* L22-9, and *P. zarinae* SWRI108. The numbers of CDSs shared or unique among different sets of strains are shown. (**d**) Position of BGCs in the genomes indicated by antiSMASH.

Comparative genomics was used to understand the evolution and to predict gene functions of the samples (Figure 4). In comparison, the genome sequence of *P. viciae* G166 was compared to the six other fully sequenced genomes of *Pseudomonas* (four type strains in the same node based on the TYGS tree and the two other *P. viciae* strains from NCBI) by MAUVE (Figure 4a). The syntenic analysis showed that horizontal gene transfer clearly emerged in the *Pseudomonas* strains. Moreover, the genomes of *P. viciae* G166 showed many regions with conserved sequences and conserved gene orders with *P. viciae* strains, except for the major inversions (Figure 4a), in accordance with the TYGS analysis (Figure 2). To identify the conserved and specific regions at the nucleotide level, the genome of *P. viciae* G166 was used as a reference genome with BLAST Ring Image Generator (BRIG) to align the genomes for *P. viciae* 11K1, *P. viciae* B21-062, *P. viciae* YsS1, *P. brassicacearum* subsp. *brassicacearum* NFM421, *P. bijieensis* L22-9, and *P. zarinae* SWRI108 (Figure 4b). The inner to outer circles depict the nucleotide position, GC content, GC skew (of *P. viciae* G166), and genes of *P. viciae* G166, *P. viciae* 11K1, *P. viciae* B21-062, *P. viciae* YsS1, *P. brassicacearum* subsp. *brassicacearum* NFM421, *P. bijieensis* L22-9, and *P. zarinae* SWRI108 (Figure 4b). The circular image depicting the comparison of the seven *pseudomonas* strains genomes revealed that the *P. viciae* G166 genome was closely related to those of the *P. viciae* strains (Figure 4b).

The genetic characteristics of *P. viciae* G166 with the four other *Pseudomonas* type strains (*P. viciae* 11K1, *P. brassicacearum* subsp. *brassicacearum* NFM421, *P. bijieensis* L22-9, and *P. zarinae* SWRI108) were further analyzed (Figure 4c, Table 2). In total, 5794, 5784, 6066, 5768, and 5936 protein-coding genes (including hypothetical proteins) of the five *Pseudomonas* strains were annotated, respectively. The predicted coding genes of all the *Pseudomonas* strains ranged from 5604 to 5784, with the G + C content ranging from 60.4 to 60.9% (Table 2). With respect to the size and GC content of the five strains, *P. viciae* G166 was more similar to *P. viciae* 11K1 (Table 2), which was confirmed by the phylogenetic relationship analysis described above (Figure 2).

A comparative analysis among the CDSs of the five *Pseudomonas* strains identified 2381 predicted protein-coding genes, which formed the core genome (Figure 4c). Except for the 2381 conserved genes shared by the four strains, *P. viciae* G166 shared 201, 142, 174, and 176 genes with *P. viciae* 11K1, *P. brassicacearum* subsp. *brassicacearum* NFM421, *P. bijieensis* L22-9, and *P. zarinae* SWRI108 (Figure 4c). Notably, there were more genes shared by *P. viciae* G166 and *P. viciae* 11K1 the other strains (Figure 4c). On the other hand, *P. viciae* G166 had 242, 311, 279, and 277 unique genes relative to genes with *P. viciae* 11K1, *P. brassicacearum* subsp. *brassicacearum* NFM421, *P. bijieensis* L22-9, and *P. zarinae* SWRI108 (Figure 4c), respectively. Furthermore, 130 unique genes were present in the genome of *P. viciae* G166.

### 3.4. Secondary Metabolite Clusters Analysis of P. viciae G166

The genome of *P. viciae* G166 was analyzed using the antiSMASH bacteria online version, and 13 secondary metabolite gene clusters were identified (Table 3). Of the thirteen clusters found, five clusters were predicted to synthesize NRPs, and the others encoded the biosynthesis of arypolyene, hserlactone, NAGGN, lantipeptide, NRP + PK, PiPP-like, NRP-like, and NI-siderophore (Table 3). Eight clusters showed similarity with the clusters known by antiSMASH. Cluster 1, belonging to NRPS-like, was similar to fragin with a 37% similarity. Cluster 2 had a 40% similarity with the cluster of arylpolyene. Clusters 5, 6, 8, 9, and 11, corresponding to non-ribosomal peptide (NRP) clusters, showed similarity with pyoverdine (10%), fengycin (13%), syringomycin (100%), thanafacin A (44%), and pyoverdine (19%), respectively. Cluster 13 was similar to the type of NRP-PK hybrids, which resembled a lankacidin cluster with a 13% similarity. The prediction of biosynthesis clusters in the six other *pseudomonas* strains was also analyzed. DAPG was predicted only in *P. brassicacearum* subsp. *brassicacearum* NFM421, and *P. bijieensis* L22-9. Based on the four secondary metabolites, *P. viciae* G166, *P. viciae* 11K1, *P. viciae* B21-062, *P. viciae* YsS1, and *P. zarinae* SWRI108 were classified as Group III *pseudomonas*. The comparison of gene clusters among the Group III *pseudomonas* strains illustrated that nine clusters (1, 2, 3, 4, 5, 6, 11, 12, and 13) with similar gene structures were shared by all the five strains (Figure 4d). Three clusters (7–9) were conserved in all the *P. viciae* strains. Based on the bioinformatic analysis, Clusters 8 and 9 were predicted to be involved in the biosynthesis of lipopeptides with similar chemical structures to the compounds produced by other *pseudomonas* (Table 3, Supplementary Figure S2). Among the NRLP clusters, Clusters 8A and 8B were related to two cyclic peptides, and Cluster 9 was related to a linear peptide. Further, Clusters 3, 4, 7, 10, and 12 were not known to have similarity with any known clusters (Table 3). A cluster for NI-siderophore was identified exclusively in *P. viciae* G166 (Figure 4d).

### 3.5. Identification and Characterization of Biocontrol Activity

**Figure 5:**
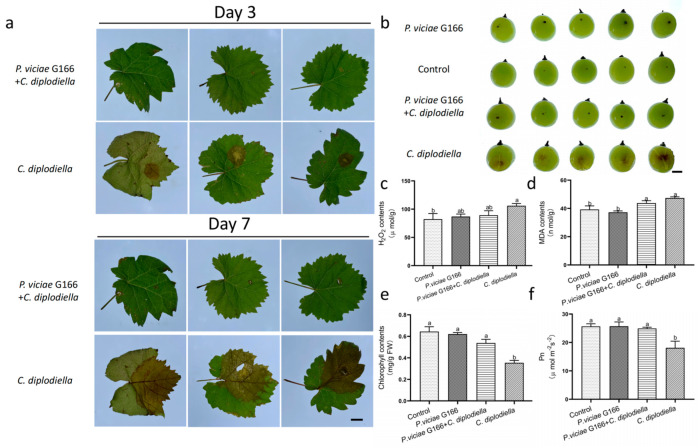
Biocontrol efficiency of G166 on grape white rot disease inoculated with *C. diplodiella.* (**a**) Symptoms inoculated with *C. diplodiella* on detached leaves either treated by G166 or not. (**b**) Symptoms inoculated with *C. diplodiella* on detached berries either treated by G166 or not. (**c**–**f**) Effects of *C. diplodiella* on MDA (**c**), H_2_O_2_ (**d**), chlorophyll contents (**e**), and net photosynthetic rate (**f**) on G166-treated plants and non-bacterized plants. *P. viciae* G166 + *C. diplodiella*, inoculated with *C. diplodiella* after inoculation with a culture of *P. viciae* G166. In (**c**–**f**), each column is the mean of four to six replicates. Bars represent standard error of the mean. Different letters above columns represent significant differences at *p* < 0.05. Scale bars: 1 cm.

To assess the biocontrol ability of *P. viciae* G166 to defend grapevine against white rot disease, detached leaves and detached berries inoculated with *C. diplodiella* on them were either subjected to or not subjected to bacterization treatment (Figure 5). Disease symptoms were evaluated by the size of necrosis. For the detached leaves, the non-bacterized treatment resulted in more disease symptoms after 3 days of inoculation with *C. diplodiella,* and the necrosis length reached 5.8 cm after 7 days of inoculation with *C. diplodiella* (Figure 5a). Compared to the non-treated leaves, the detached leaves treated by *P. viciae* G166 showed no infection symptoms after 3 days of inoculation with *C. diplodiella* and much smaller necrosis after 7 days of inoculation with *C. diplodiella* (Figure 5a). Similar to the results from the detached leaves, the G166-treated berries also demonstrated biocontrol potential against white rot disease compared to the non-treated control (Figure 5b). In detail, no case of infection occurred in the *P. viciae*-G166-treated berries under fungal treatment, while the necrosis was clear in the non-treated detached berries (Figure 5b). Clearly, these results indicate that *P. viciae* G166 remarkably reduced the severity of grape white rot disease in the grapevine.

The grapevine inoculated with *C. diplodiella* accumulated more reactive oxygen species. Excessive H_2_O_2_ accumulation can cause oxidative damage to leaves. Under the pathogen treatments, the total leaf H_2_O_2_ levels were increased by 28.9%, significantly higher than those under healthy conditions (Figure 5c). Excessive H_2_O_2_ has been shown to trigger membrane lipid peroxidation and limit membrane lipid unsaturation and membrane protein polymerization. MDA, a marker of lipid peroxidation, was increased in both the G166-pre-treated plants and the non-bacterized plants after infection by *C. diplodiella* (Figure 5d). However, it was more pronounced in the non-bacterized plants than in the G166-pre-treated plants (Figure 5d). After infection by *C. diplodiella*, the G166-pre-treated plants showed an 11.5% increase in MDA compared to the plants infected by *C. diplodiella* (20.8% Figure 5d). Oxidative damage resulted in a chlorophyll decline and subsequently decreased Pn. The chlorophyll contents decreased by 45.1% after infection by *C. diplodiella* (Figure 5e), while Pn decreased by 29.7% (Figure 5f). After infection by *C. diplodiella*, the chlorophyll contents and Pn of the plants pre-treated with *P. viciae* G166 did not significantly differ from those under healthy conditions (Figure 5e,f). Taken together, the G166-pre-treated plants exhibited protective effects in the presence of *C. diplodiell*, resulting in higher chlorophyll contents, a higher Pn, and decreased leaf H_2_O_2_ and MDA contents (Figure 5c–f).

## 4. Discussion

Grapevines suffer from many diseases that can reduce fruit productivity and affect the quality of grapes and, subsequently, the characteristics of wine. Biocontrol microbes are environmentally friendly and safe for crop production [1]. Interest in the potential of microbial inoculants for economic fruit crops has increased within the last few years. As a result, screening for new strains is a means of achieving a good level of control of a wide range of plant pathogens for fruit quality improvement. Bacteria of the genus *Pseudomonas* are ubiquitous and have high biotechnological value [10,11,12,13,14,15,38,39]. Members of *Pseudomonas* strains can be exploited in different agricultural applications such as biocontrol agents and biofertilizers [10,11,12,13]. In grapevine, *Pseudomonas* exhibited antagonistic activity and could be used as a biocontrol agent to manage GTD (grapevine trunk diseases) pathogens [40]. In this regard, discovering new rhizospheric *Pseudomonas* strains from grapevine could lead to commercial applications such as inoculants in ecological agriculture.

In this study, strain G166 was isolated from the rhizosphere of grape and exhibited broad-spectrum antagonistic activities against fungal pathogens on grapes, such as *C. diplodiella*, *B. cinerea*, and *C. gloeosporioides* (Figure 1), with mycelial growth inhibition. Here, we sequenced the entire genome of G166 and performed a comparative genome analysis (Figure 2, Figure 3 and Figure 4). The phylogenetic analysis based on the whole genome (TYGS) indicated that G166 was identified in *Pseudomonas viciae* (Figure 2). The genome of *P. viciae* G166 was estimated at 6,613,582 bp, with a GC content of 60.57% and 5749 CDSs (Table 1). Except for the genome size and GC content, the genome comparisons among the seven type strains revealed that G166 was closely related to the *P. viciae* strains (Table 2, Figure 2 and Figure 4).

As one of the common fungal diseases in China, grape white rot disease caused by *C. diplodiella* lowers grape yields and affects fruit quality [36]. The phenotype analysis showed that excessive levels of H_2_O_2_ occurred in the leaves infected by *C. diplodiella* and led to increased MDA contents (Figure 5c, d). As a result of oxidative damage, the plants exhibited a declined Pn and chlorophyll content (Figure 5e, f). Conversely, the G166-treated plants showed no significant differences in MDA contents, Pn, and chlorophyll contents after inoculation with *C. diplodiella* compared with the healthy control (Figure 5c–f). In addition, *P. viciae* G166 showed biocontrol potential against white rot disease caused by *C. diplodiella* on the detached leaves and berries, respectively (Figure 5b, c).

*P. viciae* G166 has been identified to have biocontrol potential. However, the exact mechanism of control is yet to be elucidated. The main biocontrol strategy to suppress plant pathogens is the production of secondary metabolites by *Pseudomonas*, such as 2,4-DAPG [12,16], PLT [18], PRN [17], hydrogen cyanide [41], or several peptides [19,20,21,22,23,24,25,26,27,28]. As a result, these bacteria are a prolific source of natural products. Based on conformation, the strains harbor many secondary metabolite BGCs involved in the production of NRPs, arypolyene, hserlactone, NAGGN, lantipeptide, NRP + PK, PiPP-like, and an NI-siderophore (Table 3), but not DAPG, PLT, or PRN. As a result, the *P. viciae* G166 strain is classified as a Group III *Pseudomonas* spp. based on its secondary metabolites [19]. Many isolated *Pseudomonas* spp. exhibit antimicrobial activity through the production of antimicrobial lipopeptides [19,21,22,24,25,27,28]. AntiSMASH analysis revealed five NRP gene clusters coding for the synthesis of 2 pyoverdines, fengycin, syringomycin, and thanafacin A in *P. viciae* G166 (Table 2). *P. viciae* is a novel species of the genus *Pseudomonas*. The type strain is *P. viciae* 11K1, which possesses inhibitory activity against plant pathogenic fungi and bacteria [19]. The comparative analysis revealed that Clusters 7–9 in the genome of *P. viciae* G166 showed a large degree of conservation in the *P. viciae* strains (Figure 4), indicating the possibility of the horizontal transfer of NRP biosynthesis gene clusters among *P. viciae* strains. Moreover, the structural traits of Clusters 8 and 9 were similar to those of lipopeptides exhibiting antimicrobial activity in *P. viciae* 11K1 [19]. The mechanism behind the resistances of the *P. viciae*-G166-treated grapevine against white rot disease can be explained by the secondary metabolites of Clusters 8 and 9. However, the MALDI-TOF analysis of the crude extract with antifungal activity showed that there were no identified metabolites that were the same as the molecular masses of known lipopeptides from *Psedomonas* [40]. The antifungal compounds of *P. viciae* G166 that suppress white rot disease require further investigation. Next, more research is needed to isolate and identify the bioactive natural products produced by the predicted CGSs.

In conclusion, the current study offers a comprehensive understanding of the genomic architecture and control activity of phytopathogenic fungi by the *P. viciae* G166 strain. The prediction of secondary metabolites associated with antibiosis corroborating with the experimental data suggests that the new *Pseudomonas* strain G166 can serve as a potential biocontrol agent against various diseases with agronomic importance in grapevine ecological agriculture. The comparative genomics analysis highlights increasing opportunities to discover new secondary metabolites and even new pseudomonal strains with antifungal properties. Moreover, *P. viciae* G166 can serve as a model organism for studies on the properties for grape rhizospheric colonization. Along with insights into the mechanisms of the rhizospheric colonization and the metabolites behind the biosynthesis clusters, *P. viciae* G166 may be a potential biocontrol strain and may substantiate industrialization in grapevine ecological agriculture in the near future.

## Figures and Tables

**Table 1:** General genome feathers of *P. viciae* G166.

Genome size (bp)	6,613,582
Total genes, number/total length/average length	5880/5,835,862 bp/992 bp
Gene length/genome%	88.24%
GC%	60.57%
Genes with function prediction	5749
Genes with GOs	3964
Genes connected to KEGG ontology	5663
Genes assigned to COGs	4702
CRISPR, number/total length/average length	5/915 bp/183 bp
tRNA genes, number/total length/average length	67/77 bp/5222 bp
5S rRNA genes, number/total length/average length	6/116 bp/696 bp
16S rRNA genes, number/total length/average length	5/1525 bp/7626 bp
23S rRNA genes, number/total length/average length	5/2890 bp/14,450 bp
sRNA genes, number/total length/average length	17/189 bp/3219 bp

**Table 2:** Genome characteristics of *Pseudomonas* genomes.

Genomic Features	*P. viciae* G166	*P. viciae* 11K1	*P. brassicacearum* subsp. *brassicacearum* NFM421	*P. zarinae* SWRI108	*P. bijieensis* L22-9
Size (bp)	6,613,582	6,682,832	6,843,248	6,551,245	6,730,360
GC content	60.6%	60.4%	60.8%	60.9%	60.9%
Gene count	5880	5898	6135	5825	6006
CDS count	5749	5784	6066	5768	5936

**Table 3:** General genome feathers of the G166 strain.

Cluster	Type	Size (nt)	Similar Known Cluster	Similarity
Cluster 1	NRPS-like	30,967	Fragin	37%
Cluster 2	Arylpolyene	43,611	APE Vf	40%
Cluster 3	RiPP-like	10,869		
Cluster 4	NAGGN	14,850		
Cluster 5	NRPS	51,930	Pyoverdin	10%
Cluster 6	Betalactone, NRPS	23,238	Fengycin	13%
Cluster 7	Hserlactone, NRPS	19,624		
Cluster 8	NRPS	165,451	Syringopeptin	100%
Cluster 9	NRPS	67,055	Thanafacin A	44%
Cluster 10	NI-Siderophore	18,918		
Cluster 11	NRPS	77,547	Pyoverdin	19%
Cluster 12	Lanthipeptide-class-ii	23,071		
Cluster 13	NRPS + PKS	22,165	Lankacidin C	13%

## Data Availability

The datasets presented in the study can be found online: https://www.ncbi.nlm.nih.gov/biosample/SAMN38220982/, accessed on 6 April 2024. All other data are provided in this article’s Results section and Supplementary Files.

## Supplementary Materials

The following supporting information can be downloaded at: https://www.mdpi.com/article/10.3390/jof10060398/s1, Figure S1: Phylogenetic analysis of *Pseudomonas* type strain based on 16S rDNA; Figure S2: Comparation between the predicted amino acid sequences in cluster 8 and 9 and others similar LPs; Table S1: Gene Bank accession Numbers of the strains.
