# Supplementary material for: Genome Analysis of Pseudomonas viciae G166 Conferring Antifungal Activity in Grapevine
Source: J Fungi (Basel). 2024 May 31;10(6):398. doi: 10.3390/jof10060398 (PMC11205049; doi:10.3390/jof10060398)
Supplement: Supplementary file 1 [file jof-10-00398-s001.zip › jof-2992985-supplementary.pdf]

Table S1 Gene Bank accession Numbers of the strains

Type strain	Accession Number	Assembly level
Pseudomonas aeruginosa ATCC 10145	NR_114471.1	5684 contigs
Pseudomonas aeruginosa DSM 50071	CP012001.1	Complete Genome
Pseudomonas alcaligenes ATCC 14909	NR_114472.1	Partial sequences
Pseudomonas alcaligenes IAM 12411	NR_043419.1	Partial sequences
Pseudomonas alcaligenes NBRC 14159	NZ_BATI00000000.1	122 contigs
Pseudomonas alvandae SWRI17	CP077080.1	Complete Genome
Pseudomonas bijieensis L22-9	CP048810.1	Complete Genome
Pseudomonas brassicacearum PP1_210F	NZ_AYJR00000000.1	5 Scaffolds
Pseudomonas brassicacearum DBK11	NR_024950.1	
Pseudomonas brassicacearum subsp. brassicacearum NFM421	CP002585.1	Complete Genome
Pseudomonas canavaninivorans HB002	NZ_JAEKIK000000000.1	55 Scaffolds
Pseudomonas chlororaphis ATCC 9446	CP118151.1	Complete Genome
Pseudomonas chlororaphis NBRC 3904	NR_113581.1	
Pseudomonas chlororaphis subsp. aurantiaca ATCC 33663	NR_112076.1	
Pseudomonas chlororaphis subsp. aurantiaca NCIB 10068	NR_164626.1	
Pseudomonas chlororaphis subsp. aureofaciens ATCC 13985	NR_114473.1	
Pseudomonas chlororaphis subsp. aureofaciens DSM 6698	NZ_CP027720.1	
Pseudomonas cichorii ATCC 10857	NR_112070.1	55 Scaffolds
Pseudomonas cichorii PC1	NR_026532.1	
Pseudomonas citronellolis ATCC 13674	NR_112069.1	
Pseudomonas citronellolis DSM 50332	NZ_PISI00000000.1	44 contigs
Pseudomonas citronellolis NBRC 103043	NZ_BCZY00000000.1	104 contigs
Pseudomonas corrugata CFBP2431	D87087.1	
Pseudomonas fluorescens ATCC 13525	NR_114476.1	279 contigs
Pseudomonas fluorescens CCM 2115	NR_115715.1	
Pseudomonas fluorescens IAM 12022	NR_043420.1	
Pseudomonas fluorescens NBRC 14160	NZ_BDAA00000000.1	64 contigs
Pseudomonas fragi ATCC 4973	NR_024946.1	
Pseudomonas fragi NBRC 3458	NZ_BDAB00000000.1	48 contigs
Pseudomonas fulva IAM1529	NR_115610.1	
Pseudomonas fulva NBRC 16637	NZ_BBIQ00000000.1	46 contigs
Pseudomonas fulva NRIC 0180	NR_104280.1	
Pseudomonas lini CFBP 5737	AY035996.2	
Pseudomonas lini DLE411J	NR_029042.2	1 contig
Pseudomonas marginalis ATCC 10844	LC409077.1	
Pseudomonas marginalis ICMP 3553	NR_117821.1	62 Scaffolds
Pseudomonas marginalis LMG 2210	NR_027230.1	
Pseudomonas mediterranea CFBP5447	NR_028826	
Pseudomonas mendocina ATCC 25411	NR_114477.1	
Pseudomonas mendocina NBRC 14162	NZ_BBQC00000000.1	22 contigs
Pseudomonas mendocina NCIB 10541	LC752347.1	
Pseudomonas monteilii NBRC 103158	NR_114224.1	132 contigs
Pseudomonas nitroreducens DSM 14399	NR_114975.1	50 contigs

Pseudomonas nitroreducens IAM 1439	NR_042435.1	
Pseudomonas nitroreducens NBRC 12694	NR_113601.1	40 contigs
Pseudomonas oleovorans ATCC 8062	NR_114478.1	
Pseudomonas oleovorans IAM 1508	NR_043423.1	
Pseudomonas oleovorans NBRC 13583	NR_113617.1	
Pseudomonas oleovorans subsp. lubricantis RS1	NR_115874.1	
Pseudomonas oryzihabitans IAM 1568	NR_115005.1	
Pseudomonas oryzihabitans L-1	NR_025881	
Pseudomonas oryzihabitans NBRC 102199	NR_114041.1	34 contigs
Pseudomonas protegens CHA0	CP003190.1	Complete Genome
Pseudomonas pseudoalcaligenes JCM 5968	AB021379.1	
Pseudomonas pseudoalcaligenes Stanier 63	NR_037000	
Pseudomonas putida ATCC 12633	CP101910.1	Complete Genome
Pseudomonas putida IAM 1236	NR_043424.1	
Pseudomonas rhizophila S211	CP024081.1	Complete Genome
Pseudomonas stutzeri ATCC 17588	AF094748.1	4 contigs
Pseudomonas stutzeri CCUG 11256	NR_118798.1	
Pseudomonas stutzeri NBRC 14165	AB680573.1	
Pseudomonas stutzeri VKM B-975	EU883663.1	
Pseudomonas syringae ATCC 19310	NR_114480.1	
Pseudomonas syringae DC3000	AE016853.1	Complete Genome
Pseudomonas syringae NCPPB 281	NR_043716.1	
Pseudomonas thivervalensis SBK26	NR_024951.1	
Pseudomonas tolaasii ATCC 33618	NR_114481.1	
Pseudomonas tolaasii LMG 2342	MT561438.1	
Pseudomonas viciae G166	CP146985.1	Complete Genome
Pseudomonas viciae 11K1	CP035088.1	Complete Genome
Pseudomonas viciae B21-062	CP087200.1	Complete Genome
Pseudomonas viciae YsS1	CP123771.1	Complete Genome
Pseudomonas viridiflava ATCC 13223	NR_114482.1	
Pseudomonas viridiflava CECT 458	NR_042764.1	
Pseudomonas zarinae SWRI108	CP077086.1	Complete Genome

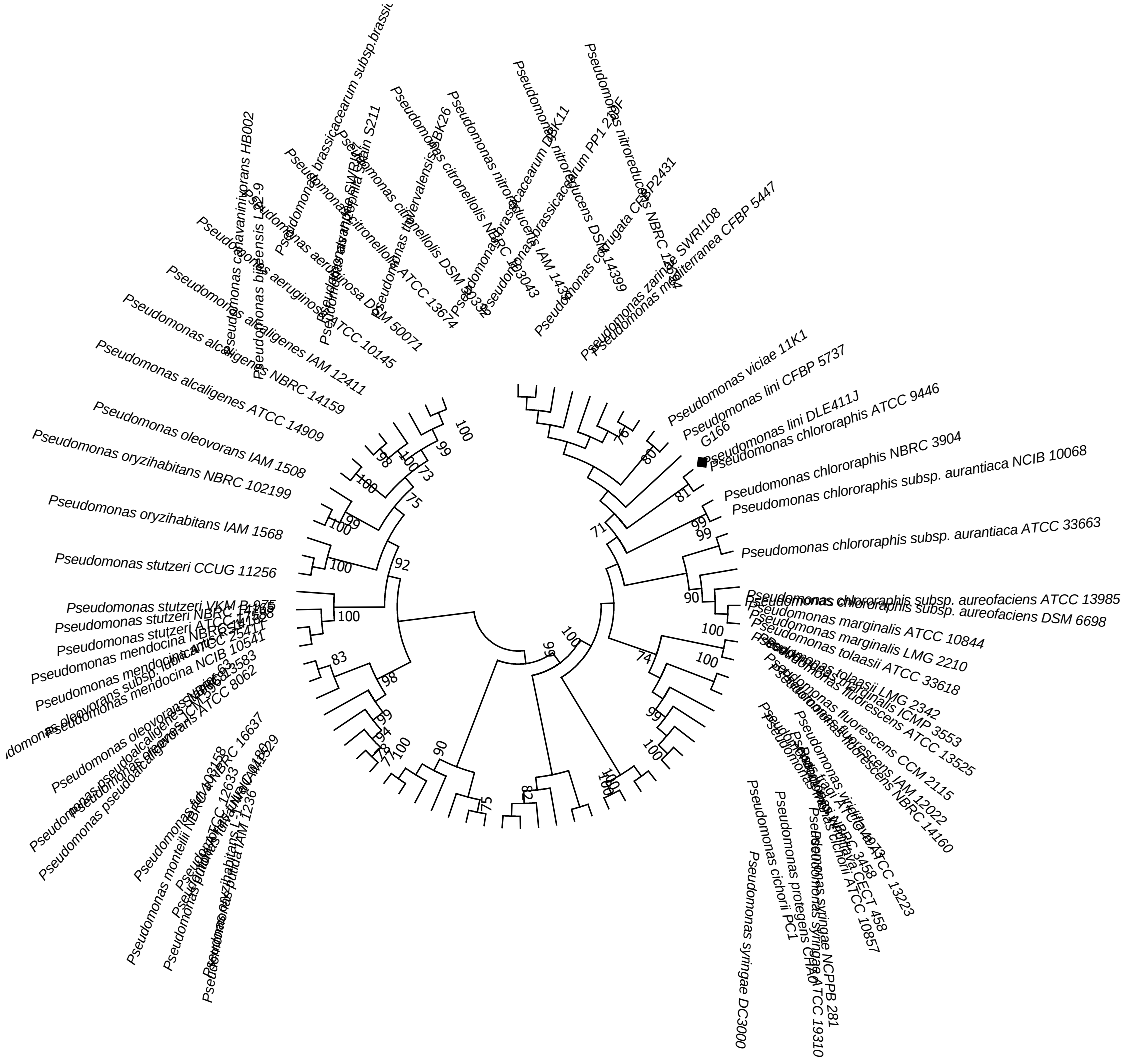

Figure S1. Phylogenetic analysis of *Pseudomonas* type strain based on 16S rDNA. Neighbor-joining phylogenetic tree based on 16S rDNA sequences showing the position of strain G166 among its phylogenetic neighbors. Phylogenetic tree of the *Pseudomonas* type strains was constructed by MEGA based on 16S rDNA using the maximum likelihood method with 1000 bootstrap replicates. Bootstraps are only indicated for branches with bootstrap support of higher than 70. Gene Bank accession Numbers are shown in Table S1.

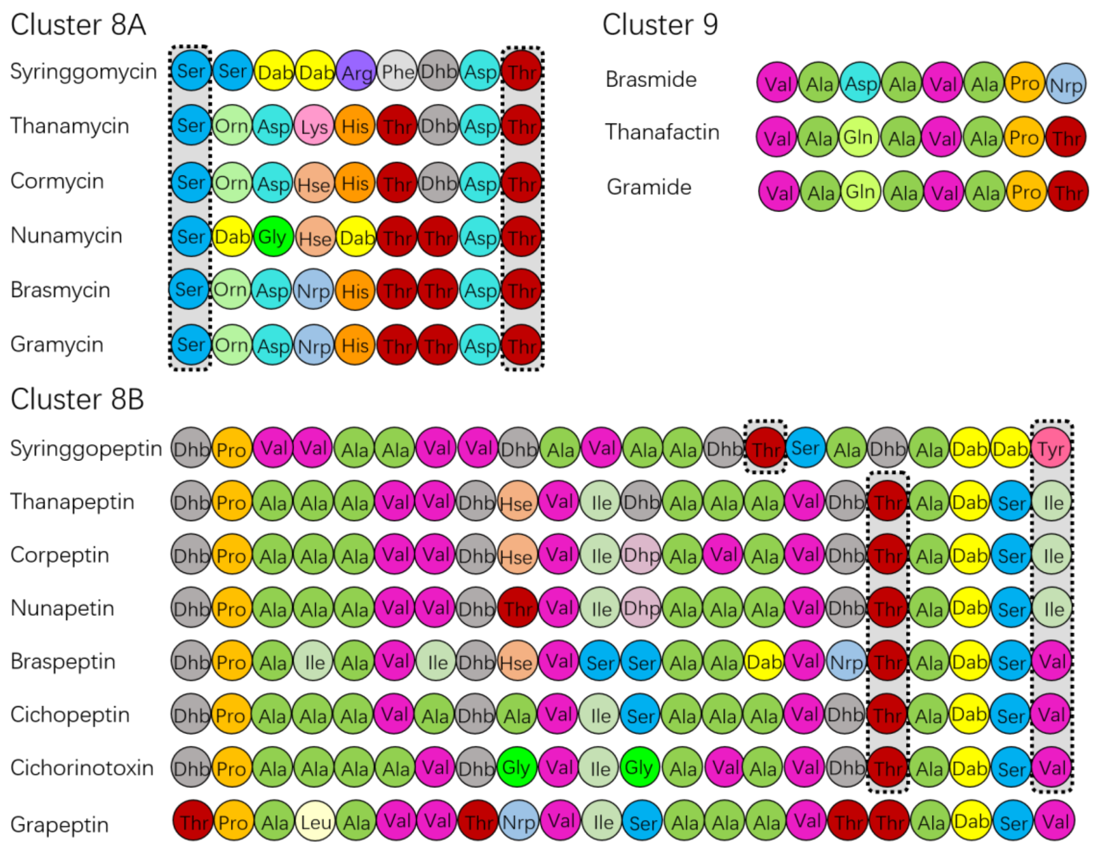

Figure S2. Comparation between the predicted amino acid sequences in cluster 8 and 9 and others similar LPs. syringomycin and syringopeptin from *P. syringae pv. syringae* B301D, thanamycin and thanapeptin from *Pseudomonas sp.* SHC52; cormycin and corpeptin from *P. corrugata* CFBP5454; nunamycin and nunapeptin from *P. fluorescens* In5; braspeptin and brasmycin from *Pseudomonas sp.* K11; cichopeptin from *P. cichorii* SF1-54; cichorinotoxin from *P. cichorii* MAFF730229. Non-standard amino acids are abbreviated as follows: Dab, 2,4-diaminobutyric acid; Dhb, dehydrobutyrine; Dha, dehydroalanine; Orn, ornithine; Hse, homoserine, Nrp, uncertain amino acids. Amino acids in the dashed boxes are responsible for the formation of the ring.
